# Is Length of Days Desirable?

**Published:** 1873-10

**Authors:** 


### IS LENGTH OF DAYS DESIRABLE?

IS A question which will be answered affirmatively, not unfrequently, by people in ill health, as well as those also who find themselves gliding along the downhill of life with little means, and with no children or friends on whose care and attention they are willing to draw for their support when they are no longer able to support themselves. Christian men, and women, too—at least, those who think themselves such—are often heard to exclaim: "I hope the Lord will call me home before I become a burden to my friends!" etc., etc. But I have generally noticed that when these same people are taken sick they are always very anxious to get well again! Let me whisper in your ears, friends, that the Lord never "called any one home" till he had lived his allotted time, which is not less than from ninety to one hundred years.

Again, this question will be answered affirmatively by all those who have a high sense of the responsibilities and the enjoyments belonging to human life, whether they be Christians or not, but more especially if they be. Are you entrusted with anything more important than your own life, reader? Precisely as you treat this important trust, then, will be its duration. It was given to you, doubtless, for your enjoyment, and why should you wish to abridge it? If existence is desirable at all, why should we not study to have the whole of it? What excuse can we make at the Judgment Day for having cut off one half, one third or one quarter of it, and that, too, the one portion of it from which we ought to derive the greatest benefit as well as enjoyment? These are serious questions, and demand the most profound consideration of the thoughtful. Do you ask, reader, what are the pleasures that belong to old age especially, and will they compensate for the physical losses? Aye, will they, and more, in all well regulated and well preserved lives.

"The view of the past," says Buffon (at seventy years of age), "offers to me the enjoyments of memory, agreeable pictures, precious images, which are more valuable than all your objects of pleasure; for these images are pleasant, pure, and call up only amiable recollections. The anxieties and remorses which belong to your youthful pleasures disappear entirely in the picture which represents them to me. It is in this way, therefore, that the moral being gains more than the physical loses."

Fontenelle, the old French philosopher, was asked, at ninety-five, which twenty years of his life he regretted the most? and his reply was, "Those between fifty-five and seventy-five."

Cornaro says: "The spirit increases in perfection as the body grows older;" and Thuseus says that "In old age attention lessens but reflection increases; this is the period in which the human heart bends back on itself and knows itself best;" and Parise, "In the old man everything is submitted to reflection."

It would appear from the testimony of these distinguished writers that old age has its own pleasures and compensations, and that it is in no sense the unenjoyable period which so many people imagine it to be. These writers, and many others, have praised it far above other periods of life for its calm and reasonable pleasures, which they found were worth living for.

With a proper regard to the laws of life and health old age is attainable by every one, unless he be the victim of accident, or, to put nearly the same thing in the words of the distinguished English scientist, Faraday, "Every death which takes place at an earlier period than ninety or one hundred is *a suicidal one.*"

With a clause in their policies forbidding the payment of losses in case of suicide, and taking Faraday's definition of the term, the life insurance business would be a paying one!

"One can hardly believe," says Parise, already quoted, "how far a little health, well treated, will carry us;" and yet who is there who has not known, in their own circle, some man or woman who has lived for a quarter of a century or more after every one supposed they were just dropping into the grave, simply by careful nursing of their own health.

A friend of ours was thought, at nineteen years of age, to be rapidly wasting away with consumption, of which he certainly had many symptoms; but he still lives in the enjoyment of good health, though that was forty odd years ago, and hopes and believes he will "still live" for the whole term of human life, and fully expects to see the new century come in!

But we have not space in the JOURNAL to elaborate these subjects as we would wish, nor would that be in accordance with our general plan; we prefer, rather, to throw out what we deem to be important suggestions, and we feel assured that all those who feel an interest in the subjects we discuss in our own way will push their investigations further in the same direction. For ourselves, we feel quite satisfied that every sincere inquirer, who studies the subject for himself, will arrive at the same conclusions that we have, viz:

1. That the natural duration of human life is nearly or quite one hundred years.

2. That this age is attainable by all who will wisely strive to secure it, and

3. That the pleasures of old age far exceed the penalties, and that, therefore, *old age is desirable.*

